# Supplementary material for: Functional hearing and low frequency hearing preservation after cochlear implant surgery is achievable with FLEX electrode arrays: Real world evidence from the MEHS Registry
Source: PLoS One. 2026 Apr 17;21(4):e0345295. doi: 10.1371/journal.pone.0345295 (PMC13089756; doi:10.1371/journal.pone.0345295)
Supplement: S1 Table — a) FLEX24 recipients b) FLEX26 recipients, c) FLEX28 recipients, d) FLEXSOFT recipients. (DOCX) [file pone.0345295.s001.docx]

**Recipients’ pre- and post-op hearing thresholds per frequency**

Table 1a: FLEX24 recipients

| **FLEX24** | **125 Hz** | **250 Hz** | **500 Hz** | **750 Hz** | **1000 Hz** | **1500 Hz** | **2000 Hz** | **3000 Hz** | **4000 Hz** | **6000 Hz** | **8000 Hz** |
| --- | --- | --- | --- | --- | --- | --- | --- | --- | --- | --- | --- |
| **N** |  |  |  |  |  |  |  |  |  |  |  |
| pre-OP | 14 | 14 | 14 | 14 | 14 | 13 | 10 | 9 | 9 | 9 | 7 |
| 6 to <12 months | 14 | 14 | 14 | 14 | 14 | 10 | 7 | 6 | 5 | 5 | 5 |
| 12 to <24months | 10 | 10 | 10 | 9 | 9 | 8 | 5 | 4 | 3 | 3 | 3 |
| 24 to <36 months | 9 | 9 | 9 | 9 | 9 | 6 | 4 | 4 | 4 | 4 | 4 |
| **Mean** |  |  |  |  |  |  |  |  |  |  |  |
| pre-OP | **19** | **23** | **45** | **66** | **81** | **96** | **104** | **108** | **107** | **97** | **92** |
| 6 to <12 months | **28** | **40** | **71** | **85** | **93** | **105** | **106** | **110** | **110** | **96** | **93** |
| 12 to <24months | **34** | **48** | **78** | **85** | **100** | **107** | **114** | **114** | **115** | **100** | **95** |
| 24 to <36 months | **32** | **49** | **81** | **92** | **98** | **106** | **120** | **120** | **115** | **100** | **95** |
| **SD** |  |  |  |  |  |  |  |  |  |  |  |
| pre-OP | 9 | 12 | 16 | 11 | 15 | 14 | 12 | 10 | 12 | 7 | 5 |
| 6 to <12 months | 16 | 19 | 25 | 18 | 14 | 9 | 10 | 7 | 9 | 9 | 4 |
| 12 to <24months | 19 | 25 | 17 | 12 | 14 | 8 | 9 | 13 | 0 | 0 | 0 |
| 24 to <36 months | 14 | 20 | 17 | 15 | 15 | 9 | 0 | 0 | 0 | 0 | 0 |

Table 1b: FLEX26 recipients

| **FLEX26** | **125 Hz** | **250 Hz** | **500 Hz** | **750 Hz** | **1000 Hz** | **1500 Hz** | **2000 Hz** | **3000 Hz** | **4000 Hz** | **6000 Hz** | **8000 Hz** |
| --- | --- | --- | --- | --- | --- | --- | --- | --- | --- | --- | --- |
| **N** |  |  |  |  |  |  |  |  |  |  |  |
| pre-OP | 4 | 4 | 4 | 4 | 4 | 4 | 4 | 4 | 4 | 4 | 4 |
| 6 to <12 months | 4 | 4 | 4 | 4 | 4 | 4 | 4 | 4 | 4 | 4 | 3 |
| 12 to <24months | 2 | 2 | 2 | 2 | 2 | 2 | 2 | 2 | 2 | 2 | 2 |
| 24 to <36 months | 0 | 0 | 0 | 0 | 0 | 0 | 0 | 0 | 0 | 0 | 0 |
| **Mean** |  |  |  |  |  |  |  |  |  |  |  |
| pre-OP | **17** | **17** | **35** | **44** | **55** | **78** | **87** | **100** | **109** | **100** | **95** |
| 6 to <12 months | **32** | **51** | **69** | **76** | **80** | **92** | **101** | **106** | **115** | **100** | **95** |
| 12 to <24months | **38** | **64** | **72** | **78** | **85** | **95** | **110** | **114** | **115** | **100** | **95** |
| 24 to <36 months | **-** | **-** | **-** | **-** | **-** | **-** | **-** | **-** | **-** | **-** | **-** |
| **SD** |  |  |  |  |  |  |  |  |  |  |  |
| pre-OP | 4 | 9 | 24 | 21 | 17 | 5 | 17 | 15 | 9 | 0 | 0 |
| 6 to <12 months | 9 | 6 | 9 | 5 | 8 | 12 | 18 | 15 | 0 | 0 | 0 |
| 12 to <24months | 25 | 20 | 16 | 11 | 7 | 0 | 14 | 9 | 0 | 0 | 0 |
| 24 to <36 months | - | - | - | - | - | - | - | - | - | - | - |

Table 1c: FLEX28 recipients

| **FLEX28** | **125 Hz** | **250 Hz** | **500 Hz** | **750 Hz** | **1000 Hz** | **1500 Hz** | **2000 Hz** | **3000 Hz** | **4000 Hz** | **6000 Hz** | **8000 Hz** |
| --- | --- | --- | --- | --- | --- | --- | --- | --- | --- | --- | --- |
| **N** |  |  |  |  |  |  |  |  |  |  |  |
| pre-OP | 48 | 48 | 48 | 48 | 48 | 48 | 48 | 46 | 43 | 41 | 40 |
| 6 to <12 months | 40 | 40 | 40 | 38 | 38 | 37 | 37 | 36 | 35 | 36 | 32 |
| 12 to <24months | 22 | 22 | 22 | 21 | 20 | 19 | 19 | 19 | 18 | 18 | 18 |
| 24 to <36 months | 10 | 10 | 10 | 10 | 9 | 6 | 6 | 6 | 6 | 6 | 5 |
| **Mean** |  |  |  |  |  |  |  |  |  |  |  |
| pre-OP | **45** | **52** | **67** | **77** | **84** | **91** | **94** | **92** | **91** | **92** | **92** |
| 6 to <12 months | **67** | **84** | **95** | **101** | **105** | **106** | **106** | **106** | **107** | **98** | **93** |
| 12 to <24months | **62** | **80** | **94** | **101** | **103** | **105** | **108** | **108** | **107** | **99** | **95** |
| 24 to <36 months | **51** | **72** | **91** | **99** | **101** | **98** | **98** | **100** | **102** | **93** | **87** |
| **SD** |  |  |  |  |  |  |  |  |  |  |  |
| pre-OP | 21 | 20 | 15 | 16 | 18 | 17 | 19 | 19 | 19 | 10 | 7 |
| 6 to <12 months | 20 | 17 | 13 | 11 | 13 | 13 | 16 | 15 | 13 | 5 | 6 |
| 12 to <24months | 21 | 20 | 18 | 13 | 14 | 12 | 13 | 15 | 13 | 3 | 0 |
| 24 to <36 months | 18 | 20 | 17 | 17 | 20 | 22 | 28 | 29 | 24 | 14 | 13 |

Table 1d: FLEXSOFT recipients

| **FLEXSOFT** | **125 Hz** | **250 Hz** | **500 Hz** | **750 Hz** | **1000 Hz** | **1500 Hz** | **2000 Hz** | **3000 Hz** | **4000 Hz** | **6000 Hz** | **8000 Hz** |
| --- | --- | --- | --- | --- | --- | --- | --- | --- | --- | --- | --- |
| **N** |  |  |  |  |  |  |  |  |  |  |  |
| pre-OP | 31 | 31 | 31 | 31 | 31 | 31 | 31 | 29 | 29 | 29 | 26 |
| 6 to <12 months | 25 | 25 | 25 | 25 | 24 | 24 | 24 | 22 | 22 | 20 | 18 |
| 12 to <24months | 12 | 12 | 12 | 12 | 12 | 12 | 12 | 11 | 10 | 8 | 7 |
| 24 to <36 months | 0 | 0 | 0 | 0 | 0 | 0 | 0 | 0 | 0 | 0 | 0 |
| **Mean** |  |  |  |  |  |  |  |  |  |  |  |
| pre-OP | **45** | **52** | **68** | **77** | **85** | **92** | **96** | **97** | **97** | **92** | **91** |
| 6 to <12 months | **80** | **94** | **101** | **107** | **113** | **113** | **114** | **114** | **112** | **100** | **95** |
| 12 to <24months | **83** | **99** | **105** | **110** | **114** | **115** | **117** | **118** | **115** | **100** | **95** |
| 24 to <36 months | **-** | **-** | **-** | **-** | **-** | **-** | **-** | **-** | **-** | **-** | **-** |
| **SD** |  |  |  |  |  |  |  |  |  |  |  |
| pre-OP | 22 | 19 | 16 | 11 | 14 | 18 | 21 | 21 | 20 | 11 | 7 |
| 6 to <12 months | 18 | 14 | 12 | 11 | 10 | 11 | 10 | 9 | 6 | 2 | 0 |
| 12 to <24months | 22 | 15 | 12 | 9 | 9 | 9 | 8 | 5 | 0 | 0 | 0 |
| 24 to <36 months | - | - | - | - | - | - | - | - | - | - | - |
