# Supplementary material for: Functional hearing and low frequency hearing preservation after cochlear implant surgery is achievable with FLEX electrode arrays: Real world evidence from the MEHS Registry
Source: PLoS One. 2026 Apr 17;21(4):e0345295. doi: 10.1371/journal.pone.0345295 (PMC13089756; doi:10.1371/journal.pone.0345295)
Supplement: S2 Table — (DOCX) [file pone.0345295.s002.docx]

|  |  | **Match: VC complete/partial LFHP & AAO functional hearing  or**  **VC complete LFHL & AAO no functional hearing** | **Mismatch: VC complete LFHL &**  **AAO functional hearing** | **Mismatch:  VC complete/partial LFHP &**  **AAO no functional hearing** |
| --- | --- | --- | --- | --- |
| **all arrays** |  |  |  |  |
| Assessments | n (%) | 89 (66.4%) | 20 (14.9%) | 25 (18.7%) |
| avg PTA3 (250, 500, 1000 Hz) VC | dB HL |  | 82.1 | 102.2 |
| avg PTA3 (125, 250, 500 Hz) AAO | dB HL |  | 62.2 | 94.0 |
| **FLEX24** |  |  |  |  |
| Assessments | n (%) | 23 (82.1%) | 4 (14.3%) | 1 (3.6%) |
| avg PTA3 (250, 500, 1000 Hz) VC | dB HL |  | 89.6 | 81.7 |
| avg PTA3 (125, 250, 500 Hz) AAO | dB HL |  | 65.0 | 91.7 |
| **FLEX26** |  |  |  |  |
| Assessments | n (%) | 2 (33.3%) | 4 (66.7%) | 0 (0.0%) |
| avg PTA3 (250, 500, 1000 Hz) VC | dB HL |  | 73.98 | - |
| avg PTA3 (125, 250, 500 Hz) AAO | dB HL |  | 57.00 | - |
| **FLEX28** |  |  |  |  |
| Assessments | n (%) | 39 (60.0%) | 11 (16.9%) | 15 (23.1%) |
| avg PTA3 (250, 500, 1000 Hz) VC | dB HL |  | 81.5 | 101.3 |
| avg PTA3 (125, 250, 500 Hz) AAO | dB HL |  | 61.8 | 92.0 |
| **FLEXSOFT** |  |  |  |  |
| assessments | n (%) | 25 (71.4%) | 1 (2.9%) | 9 (25.7%) |
| avg PTA3 (250, 500, 1000 Hz) VC | dB HL |  | 90.0 | 105.9 |
| avg PTA3 (125, 250, 500 Hz) AAO | dB HL |  | 76.0 | 97.6 |
