# Supplementary material for: Functional hearing and low frequency hearing preservation after cochlear implant surgery is achievable with FLEX electrode arrays: Real world evidence from the MEHS Registry
Source: PLoS One. 2026 Apr 17;21(4):e0345295. doi: 10.1371/journal.pone.0345295 (PMC13089756; doi:10.1371/journal.pone.0345295)
Supplement: Attachment — Submitted filename: Review PONE-D-25-39581_R1.docx [file pone.0345295.s004.docx]

**PONE-D-25-39581_R1: Low frequency hearing preservation after cochlear implant surgery is achievable with each FLEX electrode array: Real world evidence from the MEHS Registry.**

This paper presents the results of a registry study investigating the preservation of residual hearing with different cochlear implant (CI) electrodes, classified using two different methods. The strength of this approach lies in the large number of patients.

I was apparently brought in as an additional reviewer because one of the two original reviewers and the authors could not reach an agreement. Overall, the manuscript is well-written and presents relevant data. Nevertheless, I have a point of my own, which I believe needs to be added, before addressing the points of contention.

In my opinion, the statistics requested by Reviewer 2 make little sense. But what I find completely lacking is a contextualization of the results within the current literature, specifically the achieved residual hearing preservation rates for each electrode length. There are many studies where individual clinics investigate residual hearing preservation under more consistent conditions, naturally with significantly fewer patients each. Here, the rates from this large registry study need to be compared with the data from the literature.

a)

*Reviewer 2:
“Before specific points below, I recommend conducting formal statistical tests to evaluate overall/time and between-array differences and using multivariable models (with existing variables only) to identify drivers of LFHP (e.g., array length, baseline LF PTA, bilateral status, center, age/sex). Therefore, I recommend Major Revision.”*

Author’s response:
“Given that this is a registry study—with no obligation for CI users to return for follow-up visits, and with multiple outpatient facilities attended as alternative for follow up visits—we cannot ensure unbiased or continuous data collection. This would, however, be essential for robust intraindividual longitudinal analyses. Moreover, in the available registry data, variables such as electrode array length, baseline LF-PTA, and bilateral status are strongly interrelated. In addition, some key factors, such as course of deafness, hearing impairment duration, hearing aid usage prior to receiving an implant, or impedance measurements, were not included in this data set. This limits the ability to perform a multivariable model to isolate the independent effect of each variable, making robust causal or explanatory conclusions unreliable. Therefore, we believe that including such analyses would risk overstating the strength of any associations while missing out on some major factors affecting LFHP. We do not expect meaningful or clinically reliable correlations to emerge from such modeling.”

My assessment:
I agree with the authors. It is very regrettable that the dataset is not more comprehensive and has many gaps. However, this is not a prospective study with precisely controlled conditions, but rather a retrospective analysis of existing data from various clinics, which, based on experience, can vary considerably in detail. For example, I am surprised that the preoperative audiogram is up to twelve months old in some cases, and this heavily reduces the significance of the preoperative residual hearing. According to extensive literature, the duration and progression of hearing loss, hearing aid use, and deafness also have a considerable influence on the preservation of residual hearing. If this data is simply not available, it makes no sense, in my view, to feign accuracy through extensive statistics.

b)

*Reviewer 2:
“A non-trivial proportion of participants are bilateral (VC 6.1%; AAO 11.6%), raising concerns that baseline hearing severity, neuroplasticity, and ear-to-ear dependencies could yield different LFHP trajectories than in unilateral cases. Simple ear-level analyses may underestimate uncertainty if left/right ears in bilateral recipients are treated as independent. Provide a subgroup comparison of unilateral vs bilateral recipients for key endpoints (VC categories; AAO <80 dB HL) with appropriate standard errors (e.g., cluster-robust by patient). For bilateral recipients, pre-specify a consistent ear-handling rule—such as analyzing the first implanted ear only as the primary approach or using a within-patient average where justified—and include a sensitivity analysis using the alternative rule to demonstrate robustness.*

Author’s response:
“Our analytic intention, however, was not to model dependencies or to draw causal inferences about trajectories but to provide descriptive, ear-level summaries of LFHP outcomes across a broad, real-world registry. We chose ear-level as the unit of analysis because:
1. LFHP is an ear-specific phenomenon, influenced by surgical approach and array choice in that ear

2. The registry data are structured at the ear level, with no prespecified analytic framework

for handling bilateral cases. By design we only know whether a patient is bilaterally

implanted if CIs are included for both ears (thus, if the other ear had a non-MED-EL device

or was implanted in a clinic that does not participate in the registry, we would not know

that the person is a bilateral CI user),

3. From what we do know, the proportion of bilateral recipients is relatively modest (6.1%

VC; 11.6% AAO)

We consider therefore our main findings as not meaningfully altered by bilateral status or

analytic choice, while remaining aligned with the descriptive scope of our study.”

My assessment:
I agree with the authors, but I have a suggestion for further analysis, though probably only in the future when more bilateral datasets are available. The surgeon only manipulates the peripheral auditory system. Effects resulting from bilateral connections are not to be expected. The authors rightly argue that the necessary data is not available with the required clarity. This is also because it would be necessary to consider not only a cochlear implant (CI) on the contralateral side, but also residual hearing and/or hearing aid use. This data would be essential for any analysis.

Nevertheless, I find another effect very interesting. To investigate this, there are probably not yet enough datasets. If a CI is implanted on the second side, is the preservation of residual hearing comparable to that on the first side, or not? In other words, if the residual hearing on the first side is well preserved, does the patient then have a better chance on the second side? And if residual hearing is lost on the first side and no unusual occurrence is noted intraoperatively (which is naturally not recorded in the registry), does the patient then also have an increased risk on the second side? Because it may also be that factors such as predisposition or the nature of the underlying disease play a role, which have so far been recorded very little.

c)

*Reviewer 2:
“Later windows have fewer cases and potential return-bias, complicating interpretation of crosssectional proportions at 6–12/12–24/24–36 months. Fit a repeated-measures mixed-effects model for threshold change (VC bands) using all available visits. For AAO (<80 dB HL), add a timeto- event analysis (e.g., Kaplan–Meier to “loss of functional LFHP”) based on existing timestamps/interval bins, incorporating center fixed effects or a patient-level frailty term where feasible. These steps clarify temporal dynamics under missingness without requiring new data collection.*

Author’s response:
“This is a registry study and, as such, looks at real world data to provide real world evidence. As a limiting consequence, participant numbers vary across follow-up intervals.

This reflects typical clinical practice: some patients do not come back, especially if they

experience no issues. Other patients opt of follow ups at trained hearing aid centres instead of the participating clinics, as this can be more convenient in Germany.”

My assessment:
I agree with the authors. In my opinion, the purpose of the study did not require these statistics; rather, these statistics would give a false impression of accuracy.

d)

*Reviewer 2:
“FLEXSOFT shows moderate “preservation” by VC but low “functional preservation” by AAO—a clinically meaningful divergence rooted in different frequency ranges/thresholds. Where both metrics are available at the same visit, present a cross-tabulation (VC category × AAO LFHP yes/no) with agreement/discordance statistics (e.g., Cohen’s κ, McNemar’s test). Add a brief decision aid outlining when each metric should guide clinical choices (e.g., EAS candidacy versus electric-only).“*

Author’s response:

“Thanks for noticing that FLEXSOFT illustrates differing results when applying the Vienna

Consensus (VC) versus AAO metrics. However, we would like to clarify that this divergence is not clinically meaningful but rather an intrinsic feature of the two scales, which were developed to capture different aspects of hearing preservation (HP). The VC categorizes relative hearing preservation at 250, 500, and 1000 1500 Hz based on postoperative threshold shifts across a broad frequency range. Its purpose is to enable standardized, comparable reporting across centers and studies, irrespective of whether the absolute thresholds remain within a functionally usable range. The AAO metric, by contrast, is a clinically oriented framework that defines preservation as maintaining ≤80 dB HL at 125, 250, and 500 Hz—frequencies that are critical for electro-acoustic stimulation (EAS). It therefore directly addresses whether residual hearing remains of functional benefit.

Therefore, because the VC and AAO frameworks were designed to answer different questions, and because they assess different frequencies, agreement or discordance between them is neither expected nor clinically meaningful. A cross-tabulation or concordance analysis would risk suggesting comparability between two scales that are not intended to be interchangeable. Instead, their divergence simply reflects their different conceptual focus: VC quantifies relative preservation, whereas AAO defines functional preservation. (Note: This is now stated in the Methods and we have changed the 1st word of the paper’s title from “Functional” to “Low frequency”.)

This is also stressed by our results for the FLEXSOFT array—the longest in our cohort and

therefore potentially more traumatic than the shorter variants—it is expected that preservation may appear moderate under VC (reflecting limited threshold shift), while AAO indicates poor functional preservation if residual thresholds exceed 80 dB HL at low frequencies. This is not a contradiction but a natural consequence of applying two distinct scales which indeed capture different aspects of HP in a cohort of FLEXSOFT candidates who typically lack low-frequency residual hearing even pre-operatively.

In summary, the observed divergence between VC and AAO outcomes is not a clinically

meaningful inconsistency but an expected reflection of the complementary purposes of these two scales”

My assessment:
Here I find myself caught between reviewer and authors. Since both metrics (VC and AAO) have different objectives, statistics are of little use. However, I too would appreciate a descriptive comparison showing in which and how many cases the results of both metrics coincide and diverge; a table would suffice for this. The discussion should then include a paragraph that elaborates on the differences and provides an indication of which metric is better suited for which application.
