# Supplementary material for: Functional hearing and low frequency hearing preservation after cochlear implant surgery is achievable with FLEX electrode arrays: Real world evidence from the MEHS Registry
Source: PLoS One. 2026 Apr 17;21(4):e0345295. doi: 10.1371/journal.pone.0345295 (PMC13089756; doi:10.1371/journal.pone.0345295)
Supplement: Attachment — Submitted filename: Response to reviewer comments.docx [file pone.0345295.s005.docx]

EDITOR

When submitting your revision, we need you to address these additional requirements.

1. Please ensure that your manuscript meets PLOS ONE's style requirements, including those for file naming. The PLOS ONE style templates can be found at

<https://journals.plos.org/plosone/s/file?id=wjVg/PLOSOne_formatting_sample_main_body.pdf>

and

<https://journals.plos.org/plosone/s/file?id=ba62/PLOSOne_formatting_sample_title_authors_affiliations.pdf>.

The manuscript has been formatted to fit PLOS ONE.

2. Thank you for stating the following financial disclosure:

“Funding from MED-EL. There is no grant number.”

Please state what role the funders took in the study.  If the funders had no role, please state: "The funders had no role in study design, data collection and analysis, decision to publish, or preparation of the manuscript."

If this statement is not correct you must amend it as needed.

Please include this amended Role of Funder statement in your cover letter; we will change the online submission form on your behalf

The cover letter has been amended to include this information.

3. Thank you for stating the following in the Competing Interests section:

“Authors Ilona Anderson, Stefano Morettini, Karin Koinig, and Magdalena Breu are employed at MED-EL Elektromedizinische Geräte G.m.b.H.

Uwe Baumann and Daniel Polterauer have received travel support & research support from MED-EL GmbH (Austria).

Other authors disclose no conflict of interest.”

We note that one or more of the authors are employed by a commercial company: MED-EL GmbH

a. Please provide an amended Funding Statement declaring this commercial affiliation, as well as a statement regarding the Role of Funders in your study. If the funding organization did not play a role in the study design, data collection and analysis, decision to publish, or preparation of the manuscript and only provided financial support in the form of authors' salaries and/or research materials, please review your statements relating to the author contributions, and ensure you have specifically and accurately indicated the role(s) that these authors had in your study. You can update author roles in the Author Contributions section of the online submission form.

Please also include the following statement within your amended Funding Statement.

“The funder provided support in the form of salaries for authors [insert relevant initials], but did not have any additional role in the study design, data collection and analysis, decision to publish, or preparation of the manuscript. The specific roles of these authors are articulated in the ‘author contributions’ section.”

If your commercial affiliation did play a role in your study, please state and explain this role within your updated Funding Statement.

b. Please also provide an updated Competing Interests Statement declaring this commercial affiliation along with any other relevant declarations relating to employment, consultancy, patents, products in development, or marketed products, etc.

Within your Competing Interests Statement, please confirm that this commercial affiliation does not alter your adherence to all PLOS ONE policies on sharing data and materials by including the following statement: "This does not alter our adherence to  PLOS ONE policies on sharing data and materials.” (as detailed online in our guide for authors http://journals.plos.org/plosone/s/competing-interests) . If this adherence statement is not accurate and  there are restrictions on sharing of data and/or materials, please state these. Please note that we cannot proceed with consideration of your article until this information has been declared.

Please include both an updated Funding Statement and Competing Interests Statement in your cover letter. We will change the online submission form on your behalf.

The cover letter has been amended to include this information.

4. Thank you for stating the following in the Acknowledgments Section of your manuscript:

“This study was funded by MED-EL Elektromedizinische Geräte G.m.b.H. The funders assisted in study design, data collection and analysis, and preparation of the manuscript. Decision to publish was a joint decision between all authors”

We note that you have provided additional information within the Acknowledgements Section that is not currently declared in your Funding Statement. Please note that funding information should not appear in the Acknowledgments section or other areas of your manuscript. We will only publish funding information present in the Funding Statement section of the online submission form.

Please remove any funding-related text from the manuscript and let us know how you would like to update your Funding Statement. Currently, your Funding Statement reads as follows:

“Funding from MED-EL. There is no grant number.”

Please include your amended statements within your cover letter; we will change the online submission form on your behalf.

This has been deleted from the paper & the cover letter has been amended to include this information.

5. If the reviewer comments include a recommendation to cite specific previously published works, please review and evaluate these publications to determine whether they are relevant and should be cited. There is no requirement to cite these works unless the editor has indicated otherwise.

It is nice to see this, so thank you. However, in this case it is not applicable.

There are a few changes we would like to inform you of.

1.
We have updated Figures 1 and 2 to include an arrow that links the Sorting info at the top to the final *n* at the bottom, as illustrated in the screenshots below. This is the only change.

Old	New

2. We have added a reference (#11) to a recently accepted paper on this registry. This provides context but does not change content. All reference #s after 11 have been updated accordingly.
3. The corresponding author’s last name is now “Polterauer-Neuling” and not Polterauer“
4. The affiliation for ^5^ has been edited, but it is the same place.
5. Please note, the authors’ ORCIDs are:
   a. Uwe Baumann: 0000-0002-1295-2661
   b. Andreas Radeloff: 0000-0003-1881-4179
   c. Ilona Anderson: 0000-0001-7518-6661
   d. Karin A Koinig: 0000-0002-3659-4934
   e. Magdalena Breu: 0009-0007-4480-2098
   f. Stefano Morettini: 0009-0007-1568-4993
   g. Vera Lohnherr: 0000-0002-5655-7671
   h. Joachim Müller: (no ORCID)
   i. Daniel Polterauer-Neuling: 0000-0001-5008-7595

Reviewers' comments:

Reviewer's Responses to Questions

**Comments to the Author**

Reviewer #1: Thank you for the opportunity to review this manuscript and for the effort invested in conducting the study and preparing the submission. As the authors mentioned, similar papers have been published; however, the data in this study were obtained from four hospitals in Germany, which helps reduce bias related to both surgeon and patient factors. Overall, I think the manuscript to be well written and informative.

Thank you for the review & the kind words.

Minor Points

1. In the legend of Figure 2, please remove the word “right.”

Thank you, this has been corrected.

2. In Table 1, please change “gender” to “sex.”

Thank you, this has been corrected.

Reviewer #2: This multicenter registry study examines low-frequency hearing preservation (LFHP) after cochlear implantation with MED-EL FLEX arrays (FLEX24/26/28/Soft) using the Vienna Consensus (VC) and AAO metrics at 6–36 months after surgery.

The paper shows LFHP is achievable across arrays, with generally higher rates for shorter arrays and notable divergence between VC and AAO results. The topic is clinically important and valuable, and with more robust statistical analysis the manuscript would be very suitable for publication.

Before specific points below, I recommend conducting formal statistical tests to evaluate overall/time and between-array differences and using multivariable models (with existing variables only) to identify drivers of LFHP (e.g., array length, baseline LF PTA, bilateral status, center, age/sex). Therefore, I recommend Major Revision.

As stated in the manuscript, the primary aim of this data analyses was descriptive, that is to document the achievable rates of low-frequency hearing preservation (LFHP) across different MED-EL FLEX electrode arrays in a large, multicenter registry, and to compare outcomes according to two established hearing preservation definitions (Vienna Consensus and AAO). The analyses were not designed to explain longitudinal changes or to support causal inference regarding factors influencing LFHP. Accordingly, we did not conduct intra-individual longitudinal analyses, nor did we construct multivariable models for explanatory purposes.

Given that this is a registry study—with no obligation for CI users to return for follow-up visits, and with multiple outpatient facilities attended as alternative for follow up visits—we cannot ensure unbiased or continuous data collection. This would, however, be essential for robust intra-individual longitudinal analyses. Moreover, in the available registry data, variables such as electrode array length, baseline LF-PTA, and bilateral status are strongly interrelated. In addition, some key factors, such as course of deafness, hearing impairment duration, hearing aid usage prior to receiving an implant, or impedance measurements, were not included in this data set. This limits the ability to perform a multivariable model to isolate the independent effect of each variable, making robust causal or explanatory conclusions unreliable. Therefore, we believe that including such analyses would risk overstating the strength of any associations while missing out on some major factors affecting LFHP. We do not expect meaningful or clinically reliable correlations to emerge from such modeling.

Instead, the strength of our study is that it provides real-world evidence of LFHP across a spectrum of surgical practices and patient populations, while highlighting patterns of hearing preservation and differences between the Vienna Consensus and AAO definitions. We view this as a foundation on which future hypothesis-driven and longitudinal studies—designed explicitly to test causal relationships—can build.

That said, we took measures to strengthen the manuscript by:

1. Explicitly clarifying in the Introduction and Discussion that our objective was descriptive, not causal or explanatory: see lines: 83-84 and 285-290.
2. Reporting more detailed descriptive statistics in the appendix (e.g., group-level differences between arrays and over time) to aid interpretation while maintaining the descriptive scope: for see the appendix tables.
3. Highlighting in the Discussion that while multivariable modeling is theoretically possible, the current dataset and study design are not suited for robust causal inference, and that future work should address this with prospective and hypothesis-driven analyses: see lines: 291-295.

We hope this adequately addresses the reviewer’s concern while maintaining the integrity of our study design and objectives.

*Major concerns*

1. Bilateral vs unilateral patients

A non-trivial proportion of participants are bilateral (VC 6.1%; AAO 11.6%), raising concerns that baseline hearing severity, neuroplasticity, and ear-to-ear dependencies could yield different LFHP trajectories than in unilateral cases. Simple ear-level analyses may underestimate uncertainty if left/right ears in bilateral recipients are treated as independent. Provide a subgroup comparison of unilateral vs bilateral recipients for key endpoints (VC categories; AAO <80 dB HL) with appropriate standard errors (e.g., cluster-robust by patient). For bilateral recipients, pre-specify a consistent ear-handling rule—such as analyzing the first implanted ear only as the primary approach or using a within-patient average where justified—and include a sensitivity analysis using the alternative rule to demonstrate robustness.

Our analytic intention, however, was not to model dependencies or to draw causal inferences about trajectories but to provide descriptive, ear-level summaries of LFHP outcomes across a broad, real-world registry. We chose ear-level as the unit of analysis because:

1. LFHP is an ear-specific phenomenon, influenced by surgical approach and array choice in that ear
2. The registry data are structured at the ear level, with no prespecified analytic framework for handling bilateral cases. By design we only know whether a patient is bilaterally implanted if CIs are included for both ears (thus, if the other ear had a non-MED-EL device or was implanted in a clinic that does not participate in the registry, we would not know that the person is a bilateral CI user),
3. From what we do know, the proportion of bilateral recipients is relatively modest (6.1% VC; 11.6% AAO)

We consider therefore our main findings as not meaningfully altered by bilateral status or analytic choice, while remaining aligned with the descriptive scope of our study.

2. Uneven follow-up and informative missingness

Later windows have fewer cases and potential return-bias, complicating interpretation of cross-sectional proportions at 6–12/12–24/24–36 months. Fit a repeated-measures mixed-effects model for threshold change (VC bands) using all available visits. For AAO (<80 dB HL), add a time-to-event analysis (e.g., Kaplan–Meier to “loss of functional LFHP”) based on existing timestamps/interval bins, incorporating center fixed effects or a patient-level frailty term where feasible. These steps clarify temporal dynamics under missingness without requiring new data collection.

This is a registry study and, as such, looks at real world data to provide real world evidence. As a limiting consequence, participant numbers vary across follow-up intervals.

This reflects typical clinical practice: some patients do not come back, especially if they experience no issues. Other patients opt of follow ups at trained hearing aid centres instead of the participating clinics, as this can be more convenient in Germany.

3. Quantify and interpret VC vs AAO discordance

FLEXSOFT shows moderate “preservation” by VC but low “functional preservation” by AAO—a clinically meaningful divergence rooted in different frequency ranges/thresholds. Where both metrics are available at the same visit, present a cross-tabulation (VC category × AAO LFHP yes/no) with agreement/discordance statistics (e.g., Cohen’s κ, McNemar’s test). Add a brief decision aid outlining when each metric should guide clinical choices (e.g., EAS candidacy versus electric-only).

Thanks for noticing that FLEXSOFT illustrates differing results when applying the Vienna Consensus (VC) versus AAO metrics. However, we would like to clarify that this divergence is not clinically meaningful but rather an intrinsic feature of the two scales, which were developed to capture different aspects of hearing preservation (HP). The VC categorizes relative hearing preservation at 250, 500, and 1000 1500 Hz based on postoperative threshold shifts across a broad frequency range. Its purpose is to enable standardized, comparable reporting across centers and studies, irrespective of whether the absolute thresholds remain within a functionally usable range. The AAO metric, by contrast, is a clinically oriented framework that defines preservation as maintaining ≤80 dB HL at 125, 250, and 500 Hz—frequencies that are critical for electro-acoustic stimulation (EAS). It therefore directly addresses whether residual hearing remains of functional benefit.

Therefore, because the VC and AAO frameworks were designed to answer different questions, and because they assess different frequencies, agreement or discordance between them is neither expected nor clinically meaningful. A cross-tabulation or concordance analysis would risk suggesting comparability between two scales that are not intended to be interchangeable. Instead, their divergence simply reflects their different conceptual focus: VC quantifies relative preservation, whereas AAO defines functional preservation. (Note: This is now stated in the Methods and we have changed the 1^st^ word of the paper’s title from “Functional” to “Low frequency”.)

This is also stressed by our results for the FLEXSOFT array—the longest in our cohort and therefore potentially more traumatic than the shorter variants—it is expected that preservation may appear moderate under VC (reflecting limited threshold shift), while AAO indicates poor functional preservation if residual thresholds exceed 80 dB HL at low frequencies. This is not a contradiction but a natural consequence of applying two distinct scales which indeed capture different aspects of HP in a cohort of FLEXSOFT candidates who typically lack low-frequency residual hearing even pre-operatively.

In summary, the observed divergence between VC and AAO outcomes is not a clinically meaningful inconsistency but an expected reflection of the complementary purposes of these two scales

Regarding the candidacy: We would like to emphasize that neither VC nor AAO has a role in *preoperative* decision-making regarding EAS candidacy or electrode selection, as HP can only be appraised *postoperatively*, i.e., once the electrode array is already inserted. In this sense, the metrics should be viewed not as tools to guide candidacy, but rather as descriptive and evaluative measures. As such, these scales are best understood as reporting and counseling tools rather than as aids for clinical decision-making in the context of candidacy or electrode selection

*Minor concerns*

• Sample imbalance across arrays: Clearly note that FLEX28/FLEXSOFT cases dominate, reflecting practice but complicating direct comparisons across lengths; flag this in figure captions and in the Limitations.

This has been added to the limitations (lines 291-295). The N for each array is already prominently shown in Figures 3-6, thus we regard it as unnecessary to restate that there are differences in *n* in each figure caption.

• Safety summary placement: Reiterate in the Results that there was one reimplantation (cause unknown) and no device deficiencies, so safety can be grasped at a glance.

Safety has its own section in the Results: “Safety and device deficiencies”, lines 209-214.

• Uncertainty and significance in figures/tables: For all plotted means (e.g., Figures 3–6), add SEs or 95% CIs, specify which is shown, and annotate the sample size (n) at each time point and per array. Where hypothesis tests are reported, state the test/model, whether two-sided, exact p-values, and effect sizes with 95% CIs; note any multiplicity handling.

Thank you for raising this issue. We’ve added the relevant tables to the appendix for transparency. We report mean and standard deviation to highlight variability in the dataset, as we did not perform comparisons of means or medians. We chose not to include SD in the graphs to avoid visual clutter—adding error bars would obscure the changes between time points and reduce interpretability for readers.

See above, reply to earlier comment, we did not test any hypotheses with these data set.

• Define visit windows and pre-operative timing: Restate that pre-op unaided PTA was measured ≤12 months before surgery and that post-op windows are 6–12/12–24/24–36 months to aid reproducibility.

This is stated in the Methods section (lines 106-109): “For both methods, individuals were included if they had a FLEX array and unaided pure tone average (PTA) measured via air conduction at ≤12 months preoperatively and at least one assessment 6–36 months postoperatively.”.

• Per-ear reporting clarity: Add “per ear” to figure/table titles and direct readers to the bilateral-handling rule in Methods.

Per ear has been added to all tables/table captions and to the captions in figures 3-6.
